# Kinematic Analysis of Gait in the Second and Third Trimesters of Pregnancy

**DOI:** 10.1155/2013/718095

**Published:** 2013-01-31

**Authors:** Marco Branco, Rita Santos-Rocha, Liliana Aguiar, Filomena Vieira, António Veloso

**Affiliations:** ^1^CIPER, Faculdade de Motricidade Humana, Estrada da Costa, Dafundo, 1495-688 Cruz Quebrada, Portugal; ^2^ESDRM-IPS, Escola Superior de Desporto de Rio Maior, Avenida Dr. Mário Soares, 2040-413 Rio Maior, Portugal; ^3^FMH-UTL, Faculdade de Motricidade Humana, Estrada da Costa, Dafundo, 1495-688 Cruz Quebrada, Portugal

## Abstract

The kinematic analysis of gait during pregnancy provides more information about the anatomical changes and contributes to exercise and rehabilitation prescription. The purposes were to quantify the lower limb kinematics of gait and to compare it between the second and third trimesters of pregnancy and with a control group. A three-dimensional analysis was performed in twenty-two pregnant women and twelve nonpregnant. Repeated Measures and Manova tests were performed for comparisons between trimesters and between pregnant and controls. The walking speed, stride width, right-/left-step time, cycle time and time of support, and flight phases remain unchanged between trimesters and between pregnant and controls. Stride and right-/left-step lengths decreased between trimesters. Double limb support time increased between trimesters, and it increased when compared with controls. Joint kinematics showed a significant decrease of right-hip extension and adduction during stance phase between trimesters and when compared with controls. Also, an increase in left-knee flexion and a decrease in right-ankle plantarflexion were found between trimesters. The results suggested that pregnant women need to maintain greater stability of body and to become more efficient in locomotion. Further data from the beginning of pregnancy anthropometric data may contribute to the analysis.

## 1. Introduction

The third trimester of pregnancy is characterized by a rapid growth in size and weight of the fetus, so that an additional 50% increase in fetal weight is observed in this trimester [1]. This in turn causes an increase in abdominal weight and volume in pregnant woman, which is associated to an increase in the weight of growing breasts and an increase in lumbar lordosis, resulting in a superior and posterior shifts of the woman's center of gravity [2]. These changes, occurring in the body of the pregnant woman, lead to many complains of discomfort and pain in lower limbs. Few studies describe the kinematic motion on the lower limb of the pregnant women, particularly in a longitudinal perspective.

In previous studies, Foti et al. [3] performed a three-dimensional (3D) analysis of gait during the second half of the last trimester of pregnancy and one year postpartum on 15 women. They reported that overall gait kinematics were unchanged during pregnancy. However, significant increases in hip and ankle kinetics were found. Their findings indicate that during pregnancy there may be an increased demand placed on hip abductor, hip extensor, and ankle plantar flexor muscles during walking. Lymbery and Gilleard [4] investigated the temporospatial and ground reaction forces (GRF) variables in the stance phase of walking during late pregnancy of 13 women at 38 weeks' gestation and 8 weeks after birth. They concluded that in late pregnancy, there was a wider step width, and mediolateral GRF tended to be increased in a medial direction. They suggested that women may adapt their gait to maximize stability in the stance phase of walking and to control mediolateral motion. Huang et al. [5] compared the natural pattern of walking of 10 nulligravidae and 10 pregnant women, divided into three groups, respectively, at 12 weeks, at 13–28 weeks, at 29–40 weeks of gestational age and tested only one time. They reported significant differences between the pregnant and nonpregnant women, especially in knee abduction angle, knee and hip internal rotation angles. Also, as gestational age increases, the experimental group increased hip extension moment, decreased knee extension moment, increased knee adduction moment, and decreased ankle plantar flexion moment, and these changes were related with sacroiliac pain. The authors suggested that the hip is the main work-loading area. Little published data in this field analyzed what changes occur in each trimester or associated the kinematic and kinetic variables for each phase of the gait cycle. Other reason to study the gait as pregnancy progresses is to associate the gait variables with the increased prevalence of back and foot pain and other clinical complications. Also, the analysis of both sides of the body might be interesting in order to understand potential imbalances.

The purpose of this study was to describe spatial and temporal parameters and quantify the kinematic variables on the structures of the lower limb during gait and compare it between the later stages of second and third trimesters of pregnancy and with a control group of nonpregnant women.

## 2. Materials and Methods

### 2.1. Participants

Twenty-two pregnant women, between the ages of 27 and 38 years and with no history of foot, ankle, knee, musculoskeletal, and neuromuscular trauma or disease, participated in this study. Pregnant participants were recruited via direct contact and flyers placed in gym and health centers and have volunteered to participate in the study. Twelve healthy nulligravidae women participated in the study as controls. None of the participants had contraindication to physical exercise. All subjects gave written informed consent prior to participation in the study.

The pregnant participants presented the following characteristics: mean (± sd) age of 32.5 ± 2.6 years (range: 27.0–38.0); height of 1.62 ± 0.06 m (range: 1.50–1.76); number of gestational weeks of 27.0 ± 1.3 weeks (range: 25.0–29.1) in the second trimester (2T); mass of 67.1 ± 6.9 Kg (range: 55.5–85.0) in the 2T; body mass index (BMI) of 25.6 ± 2.9 Kg/m^2^ (range: 21.4–33.2) in the 2T; number of gestational weeks of 36.3 ± 1.0 weeks (range: 34.6–38.4) in the third trimester (3T); mass of 71.4 ± 6.7 Kg (range: 59.0–87.0) in the 3T; BMI of 27.3 ± 2.8 Kg/m^2^ (range: 22.8–34.0) in the 3T.

The nonpregnant group presented the following characteristics: mean (± sd) age of 20.58 ± 1.73 years (range: 18.0–23.0); height of 1.64 ± 0.07 m (range: 1.54–1.73); mass of 58.33 ± 8.71 Kg (range: 45.0–73.5); BMI of 21.5 ± 2.4 Kg/m^2^ (range: 18.1–25.7).

### 2.2. Procedures prior to Motor Task

The study was approved by the ethical committee of the faculty, and data were collected at the Laboratory of Biomechanics and Functional Morphology, in two times: during the later stages of the second trimester (2T) and third trimester (3T). 

Before performing the motor task, anthropometric data was measured, according to the International Society for the Advancement of Kinanthropometry (ISAK) standardized measurement protocol [6] by ISAK certified anthropometrists, with exception of the abdominal girth [7].

**Figure 1:**
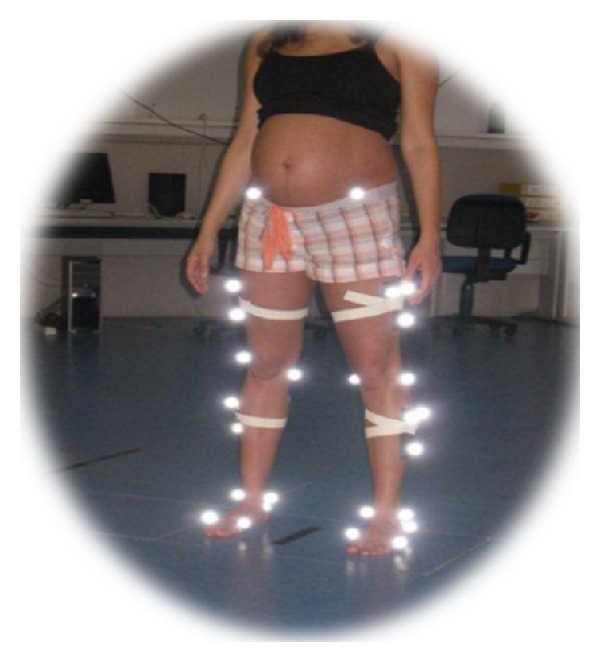
Spherical reflective markers placed with double-sided adhesive tape on the skin, in both sides of the lower body of a pregnant women.

In order to collect static and dynamic data trials, spherical reflective markers were placed with double-sided adhesive tape on the skin, in both sides of the lower body (Figure 1).

Markers setup is in agreement with the suggestion of Cappozzo et al. [8], for lower limb segments, and CODA (Charnwood Dynamics Ltd, Leicestershire, United Kingdom) protocols for model of pelvis segment. Thereby, in the static trial, for foot model, markers were placed on the fifth metatarsal head, first metatarsal head, posterior proximal top of calcaneus, posterior distal top of calcaneus, lateral top of calcaneus. For shank model construction, markers were placed on the lateral malleolus, medial malleolus, lateral femoral epicondyle, medial femoral epicondyle, and a cluster with 3 markers in the lateral of shank. For the thigh model construction, markers were placed on the lateral femoral epicondyle, medial femoral epicondyle, and a cluster with 3 markers in the lateral of thigh. To define the CODA pelvis model, markers were placed in both anterior superior iliac spine and posterior superior iliac spine. For the dynamic trials, at least 3 markers were left in each segment, as reference to static markers setup. Planar motion of the hip, knee, and ankle joint was calculated with Visual 3D software (C-Motion Inc., Germantown, USA) by a computational procedure implementing the dot product between the skeletal segments articulated by these joints.

### 2.3. Motor Task

The motor task was to walk barefoot a distance of 10 meters between two points, in a straight line at a natural and comfortable speed, as suggested in previous studies [5, 9], for 3 minutes, with a time break of 1 minute between each trial. The floor had no specific patterns or irregularities, and the participants had no knowledge of the location of force platforms. Participants were allowed to get familiar with the laboratory system, and no fatigue occurrence was reported.

### 2.4. Kinematic Data Collection

Kinematic data were collected through ten infrared high-speed cameras (Oqus-300, Qualisys, Sweden) at a rate of 200 Hz and two Kistler force platforms (Kistler AG, Winterthur, Switzerland) of 0.60 m × 0.40 m (length, width), at a rate of 1000 Hz. The capture hardware was connected to Qualisys USB Analog Acquisition interface in order to synchronize kinetic and kinematic data with software Qualisys Track Manager (QTM; Qualisys AB, Gothenburg, Sweden). Data sequences, of cameras and force platforms, were recorded in the same file. System was calibrated by wand type, with an exact wand length of 751.4 mm moved randomly across the recorded field, before each participant data collection. Calibration was accepted if the standard deviation of the wand's length measures was below 0.5 mm. Cameras were positioned statically to minimize light reflection artifacts and to allow recording of at least two consecutive walking cycles, defined as the time between two consecutive initial ground contacts of the heel strike for each side. The last cycles performed by each participant were considered for the analysis. Digital images (of the markers) were collected at same time as the GRF. 

### 2.5. Kinematic Data Analysis

A three-dimensional (3D) analysis was performed including both sides of the body and also in the transverse plane. Gait events and walking cycles were manually defined based on the vertical trajectory of the proximal end of the foot segment and on the vertical GRF curve. Collected data were interpolated using a Cubic Spline Interpolation as suggested by Robertson et al. [10], for a maximum of 10 frames gap. The trajectory of the reflective markers and the kinetic data were filtered with a Butterworth digital lowpass filter, at 10 Hz cutoff frequency, as suggested by Robertson and Dowling [11]. All data were normalized in time.

Considering the two trimesters in analysis, kinematic pattern curves (angular displacement in ankle, knee and hip in degrees) were estimated relative to the walking stride cycle. The data curves and the peak angles values were estimated, for left and right side, with visual 3D. The mean and standard deviations were analyzed in IBM SPSS Statistics (version 20). The range of motion of each joint was also analyzed in IBM SPSS Statistics (version 20). For kinematic and kinetic parameters, initial foot contact was collected at the time corresponding to first contact of the foot on the floor. For kinematic parameters, the end of the stride corresponded to the next contact with the same foot. Four strides of each subject were considered in the two trimesters. 

### 2.6. Variables Analyzed

The independent variables were the 2T and 3T. The following dependent variables were analyzed:

- walking speed;
- cycle time;
- right- and left-step time;
- double limb support time;
- time of support and flight phases in both lower limbs;
- stride width;
- stride length;
- right- and left-step length;
- joint angles in the sagittal plane of the hip, knee, and ankle for right and left lower limbs;
- in the *hip* joint two peaks were considered in the *sagittal* plane: the first peak represents the maximum hip extension which occurs in the toe off event; the second peak occurs some instants before heel strike and represents the maximum hip flexion;
- in the *knee* joint four peaks were considered in the *sagittal* plane: the first peak occurs after the heel strike and represents a slightly flexion to absorb the contact with the floor; the second peak represents the slight knee extension near the late mid stance phase; the third peak occurs in the mid swing phase as the maximum knee flexion; and the fourth peak, represents the maximum extension of the knee and occurs instantly before heel strike;
- in the *ankle* joint four peaks were considered in the *sagittal* plane: the first peak occurs immediately after heel strike with a sudden decrease of dorsiflexion of the foot; the second peak occurs approximately at contralateral heel strike; the third peak indicates the maximum plantarflexion at toe off event with a decrease of plantarflexion; the fourth peak occurs in mid swing phase and represents the maximum dorsiflexion of the foot in preparation for contact with the ground;
- joint angles in the frontal plane of the hip and ankle for right and left lower limbs;
- in the *hip* joint two peaks were considered in the *frontal* plane: the first peak occurs in the mid-stance phase and represents the maximum value of hip adduction; the second peak occurs after toe off event and represents the maximum value of hip abduction;
- in the *ankle* joint four peaks were considered in the *frontal* plane: the first peak occurs in mid stance in the maximum ankle eversion phase; the second peak represents the maximum value of ankle inversion during toe off event; the third peak occurs in the mid swing phase close to the neutral position; the fourth peak occurs instantly at the end of the swing phase in inversion;
- joint angles in the transverse plane of the hip and ankle for right and left lower limbs;
- in the *hip* joint two peaks were considered in the *transverse* plane: the first peak represents the maximum value of internal rotation, and it occurs instantly before the toe off event; the second peak represents the maximum value of external rotation, and it occurs in a late swing phase;
- in the *ankle* joint two peaks were considered in the *transverse* plane: the first peak occurs at the beginning of the mid-stance phase, and the second peak occurs in the mid swing phase;
- kinetic pattern of gait-ground reaction forces (GRF).

The determination of angle peaks was performed according to Rose and Gamble [12]. However, few more peaks were also included in the analysis: in the sagittal plane, two more peaks in the knee were included, which represent the extension of the shank. In the frontal plane, four peaks were calculated in the ankle joint. The first peak occurs between the heel strike and the contralateral foot toe off and represents the largest peak of the foot eversion. The second peak occurs immediately before the toe off of the first foot and represents the highest peak of the foot inversion. The third peak occurs during the swing phase of the first foot and represents the returning to the neutral position, and, finally, the fourth peak occurs immediately to heel strike and represents an inversion peak. In the transverse plane, two peaks were calculated at the ankle joint. Both peaks represent an abduction of the foot. The first peak occurs after the heel strike, and the second occurs during the swing phase.

### 2.7. Statistical Procedures

All statistical procedures were conducted using IBM SPSS Statistics (version 20) software for Windows. Shapiro-Wilk normality test was conducted and not assumed for all cases. The Mauchly's test of sphericity was performed before Repeated Measures analysis and was assumed. For pairwise analysis, the Repeated Measures analysis was performed between second and third trimesters. All the requirements for application of the Repeated Measures and MANOVA analyses were calculated and assumed. MANOVA was applied between each of the trimesters and the group of nonpregnant, to verify what was the level of change between nonpregnant and the pregnant participants. Bonferroni confidence interval adjustment was applied to allow an adjustment to the confidence intervals and significance values for multiple comparisons. As suggested by Vincent [13], for all cases, the level of statistical significance was set at *P* ≤ 0.05.

## 3. Results 

### 3.1. Spatiotemporal Parameters

Spatiotemporal data are presented in Table 1. After performing the Repeated Measures analysis between the second and third trimesters, it was found that the results are influenced by the effect of the trimester to which they relate. However, most of the spatiotemporal parameters remain unchanged between trimesters. Thereby no significant differences were found in walking speed, stride width, right and left step time, cycle time, and in the time of support and flight phases in both lower limbs. Significant differences were observed in right- and left-step length, stride length that decreased from 2T to 3T, and in double limb support time that increased between trimesters (*F* = 122.342, *P* = 0.000; power = 0.853). Among these variables there was no difference between the left- and right-step length variables, pointing out that although there are differences between trimesters these differences do not occur laterally.

The MANOVA analysis between the second trimester and the group of nonpregnant women has shown that there was no influence between experimental variables, and the results are dependent on the trimester which they relate. However, between the group of nonpregnant and pregnant women in the second trimester, differences were observed in double support time. The same analysis between the group of nonpregnant and pregnant women in the third trimester also has shown that there was no influence between experimental variables, and the results are dependent on the group they belong to. Significant differences were found in the stride length, in the right- and left-step length and in double support time.

### 3.2. Joint Kinematics

The joints range of motion was analyzed in all motion planes, and data are presented in Tables 1, 2, and 3.

### 3.3. Joint Kinematics: Sagittal Plane

**Figure 2:**
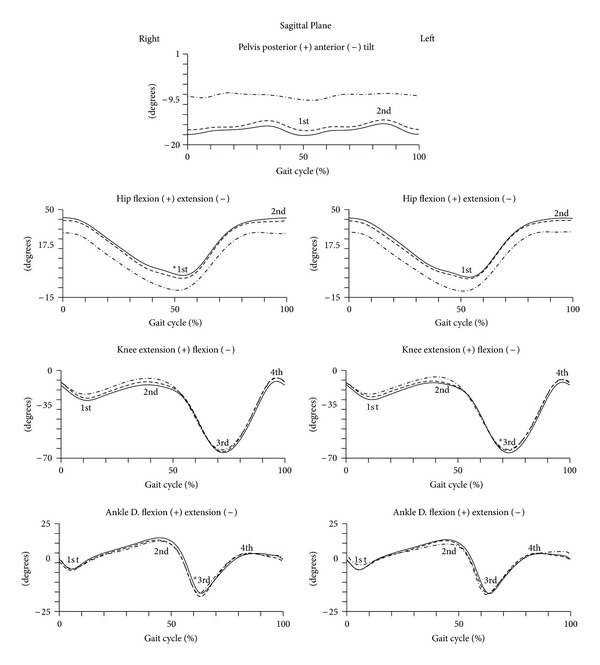
Kinematic parameters (sagittal plane) of gait of pregnant women in the later stages of second trimester (dashed line) and third trimester (solid line) and of nulliparous controls (dot line). Mean joint angles of the pelvis, hip, knee, and ankle, for right and left lower limbs, in degrees. The curve peaks are indicated by numbers: first, second, third and fourth and (*) points the significant differences.

The kinematic pattern of the gait in sagittal plane is represented in Figure 2. The quantitative data are presented in Table 2.

The Repeated Measures analysis has shown that the angular data are dependent on pregnancy trimester, and there was no angular dependence between angle peaks. The first peak of the hip joint, presented in the sagittal plane, a significant decrease in its magnitude, keeping the thigh close to the neutral position at the end of the stance phase (*F* = 6.390; *P* = 0.001; power = 0.233). The peaks of the knee joint remain with similar magnitude from the second to the third trimester; however, the third peak performs a significant increase of 1.2 degrees of knee flexion during the swing phase. The analysis of the angular displacement between second and third trimesters showed that most of the peaks angles of the ankle remain unchanged. However, the third peak of the right ankle has shown a significant reduction of its angular magnitude, signifying a decrease in plantar flexion performed in the third trimester, of about 1.4 degrees. The remaining peaks did not show significant changes between trimesters. 

In multivariate analysis between the group of nonpregnant and pregnant women in the second trimester, there were significant differences only in the first peak of the hip joint (*F* = 18.697; *P* = 0.000; power = 0.369). Between the group of nonpregnant and those in the third trimester, in the sagittal plane, also significant differences in the first peak of the hip joint (*F* = 36.922; *P* = 0.000; power = 0.536) were found.

### 3.4. Joint Kinematics: Frontal Plane

**Figure 3:**
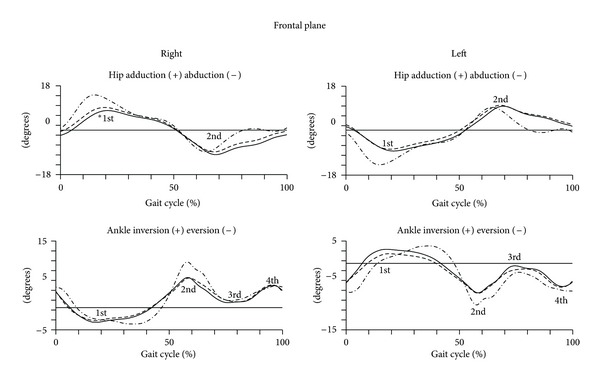
Kinematic parameters (frontal plane) of gait of pregnant women in the later stages of second trimester (dashed line) and third trimester (solid line) and of nulliparous controls (dot line). Mean joint angles of the hip and ankle, for right and left lower limbs, in degrees. The curve peaks are indicated by numbers: first, second, third and fourth and (*) points the significant differences.

The kinematic analysis of the gait in frontal plane is represented in Figure 3. The quantitative data are presented in Table 3.

The Repeated Measures analysis of the angular displacement of the second to the third trimester of pregnancy, in the frontal plane, revealed that the results are dependent on the trimester to which it relates. However, in the hip joint there was a significant change in the first peak, which represents a decrease of magnitude of abduction of the thigh of about 1.4 degrees from the second to the third trimesters. The same peak revealed differences between the group of nonpregnant and the pregnant women in the second trimester (*F* = 5.412; *P* = 0.026; power = 0.145) and the women in late pregnancy (*F* = 12.876; *P* = 0.001; power = 0.287). In the ankle there were no significant changes in angular peaks.

### 3.5. Joint Kinematics: Transverse Plane

**Figure 4:**
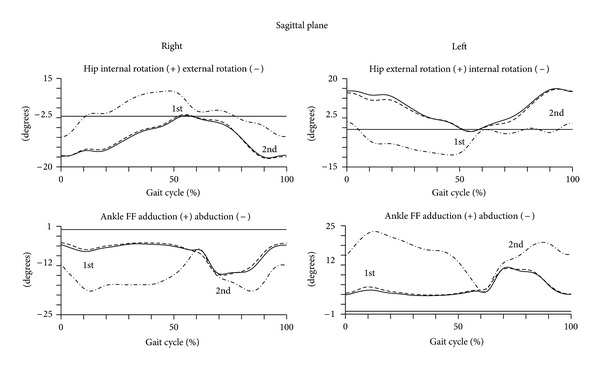
Kinematic parameters (transverse plane) of gait of pregnant women in the later stages of second trimester (dashed line) and third trimester (solid line) and of nulliparous controls (dot line). Mean joint angles of the hip and ankle, for right and left lower limbs, in degrees. The curve peaks are indicated by numbers: first and second and (*) points the significant differences.

The kinematic analysis of the gait in transverse plane is represented in Figure 4. The quantitative data are presented in Table 4. 

In the transverse plane, the ankle, knee, and hip joints have shown no significant changes between the second and third trimesters and also no changes between pregnant and control group.

## 4. Discussion

The knowledge of the kinematic parameters associated to gait and other motor tasks performed by the pregnant woman, during the three trimesters of pregnancy and postpartum, provides more information about the effect of pregnancy in a range of performance conditions. This kind of information will be helpful for prescribing exercise programs and rehabilitation programs and preventing musculoskeletal injuries.

The unchanged results found in most temporal parameters of walking in the third trimester are similar to results found by Foti et al. [3] and Lymbery and Gilleard [4]. Our results showed an increased time of double support between the group of nonpregnant and pregnant women and between the second and third trimesters. Similar results were also found by Foti et al. [3]. Furthermore, in spatial parameters, it was found a significant decrease in the length of right and left step and therefore the size of the gait stride, from the nonpregnant group and the second trimester of pregnancy. Both spatial and temporal parameters corroborate that, while walking at a self-selected pace, the pregnant woman needs to promote stability of the body. The observed decrease in stride length, while the double limb support time increased, between the second and third trimesters, might be related to the fact that pregnant women experience an altered eye contact with the floor due to abdomen volume. Also, if the pregnant women became heavier by the end of their pregnancy, they are supposed to be more careful when walking to protect themselves from falling and possibly injuring the fetus.

In the analysis of joint kinematics, the range of motion in the transverse plane of the right hip was the only parameter to experience significant changes, with a reduction in its amplitude, possibly due to increased volume in the abdominal region or to the lateral dominance; however, this data were not collected. 

The analysis of angular peaks revealed that most of the peaks remain unchanged during pregnancy. However, between trimesters significant differences were found in the extension and abduction peaks of the right thigh, in the maximum flexion peak of the left knee, and in the plantarflexion peak of the right ankle. When those peaks were compared between nonpregnant group and the groups in both trimesters, there was a significant reduction in the extension and abduction of the right thigh. These results highlight that the hip joint, possibly because it is near the pelvic region, carries more angular adjustments, especially during the stance phase.

Considering the variables analyzed, in the majority, the same behavior was observed in right and left lower limbs. Differences between right and left sides of the body were not expected. However, few differences that were found between both sides and the potential imbalances related to these cases, need further analysis.

## 5. Conclusion 

In conclusion, considering all planes of motion we find that most of the studied parameters remain unchanged between the second and third trimester of pregnancy. However, parameters related to the stance, and corresponding time, suggested that participants need to maintain greater stability of body. Nevertheless, it may induce discomfort and pain in the lower limbs often reported by pregnant women. These changes also may promote the pregnant women to become more efficient in locomotion. Much of the differences found during pregnancy are dependent to which trimester they belong, and we believe that these changes may happen from the beginning of pregnancy to the end of it with greater magnitude, because when compared with the group of nonpregnant, greater magnitude of differences were verified. However, further data from the beginning of pregnancy are required. The inclusion of anthropometric data may also contribute to the analysis of its influence on biomechanical parameters. The literature primarily analyzes the changes between the end of pregnancy to postpartum; however, it may be wise to assume that pregnancy induces changes that remain in the postpartum period, in a way justifying that much of the studied parameters remain unchanged as was reported by Foti et al. [3]. Further data from the beginning of pregnancy are needed, and also the inclusion of anthropometric data may also contribute to the analysis of gait during pregnancy and its influence on biomechanical parameters.

## Figures and Tables

**Table 1:** Spatiotemporal parameters of gait during the later stages of the second and third trimesters of pregnancy (*N* = 22) and nulliparous controls (*N* = 22). Units (mean ± sd) of mass (Kg), velocity (m/s), length (m), and time (s).

	Second trimester (2T)	Third trimester (3T)		Nonpregnant (NP)	
Mass (Kg)	**67.082 ± 6.946**	**71.368 ± 6.652**	↑	58.333 ± 8.711	
Velocity (m/s)	1.159 ± 0.125	1.127 ± 0.128		1.243 ± 0.089	
Stride width (m)	0.096 ± 0.025	0.101 ± 0.027		0.078 ± 0.024	
Stride length (m)	**1.260 ± 0.098**	**1.234 ± 0.088**	↓	**1.316 ± 0.099**	^3^↑
Left-step length (m)	**0.630 ± 0.051**	**0.616 ± 0.044**	↓	**0.657 ± 0.053**	^3^↑
Right-step length (m)	**0.630 ± 0.049**	**0.618 ± 0.045**	↓	**0.659 ± 0.049**	^3^↑
Cycle time (s)	1.081 ± 0.054	1.091 ± 0.063		1.048 ± 0.040	
Left-step time (s)	0.541 ± 0.030	0.545 ± 0.032		0.523 ± 0.020	
Right-step time (s)	0.540 ± 0.026	0.545 ± 0.033		0.525 ± 0.021	
Left-stance time (s)	0.640 ± 0.040	0.655 ± 0.046		0.616 ± 0.026	
Left-swing time (s)	0.439 ± 0.018	0.435 ± 0.021		0.433 ± 0.018	
Right-stance time (s)	0.645 ± 0.042	0.656 ± 0.042		0.616 ± 0.029	
Right-swing time (s)	0.438 ± 0.019	0.436 ± 0.025		0.433 ± 0.020	
Double limb support time (s)	**0.208 ± 0.029**	**0.219 ± 0.030**	**↑**	**0.183 ± 0.018**	^2,3^↓

Bold: significant differences with *P* < 0.05.

^3^↑significance only with third trimester.

^2,3^↓ significance with second and third trimesters.

**Table 2:** Joint kinematic peak values (mean) of gait in the sagittal plane, during the second and third trimesters of pregnancy (*N* = 22) and nulliparous controls (*N* = 12). Units (mean ± sd) are in degrees.

Joint	Side	Peak	Second trimester	Third trimester	Nonpregnant
Pelvis		1st	−14.874 ± 3.653	−15.195 ± 4.141	−10.355 ± 2.879
		2nd	−18.036 ± 3.566	−18.706 ± 3.523	−7.127 ± 2.515
Hip	Right	1st	−2.301 ± 5.282	−0.295 ± 4.507	−10.072 ± 4.437
		2nd	41.510 ± 4.413	43.168 ± 3.897	34.030 ± 2.998
	Left	1st	−2.903 ± 6.347	−1.963 ± 5.150	−10.710 ± 4.388
		2nd	41.853 ± 3.793	43.628 ± 3.488	34.498 ± 2.786
Knee	Right	1st	−16.427 ± 6.689	−18.175 ± 7.152	−18.862 ± 5.770
		2nd	−2.620 ± 5.161	−4.464 ± 6.521	−5.861 ± 4.825
		3rd	−60.516 ± 14.282	−61.864 ± 14.219	−62.815 ± 5.575
		4th	−1.035 ± 5.535	−3.350 ± 7.188	−5.879 ± 5.262
	Left	1st	−16.402 ± 6.651	−17.665 ± 7.478	−19.248 ± 6.194
		2nd	−2.444 ± 7.224	−3.091 ± 7.028	−5.297 ± 4.281
		3rd	−63.781 ± 3.828	−65.074 ± 3.441	−63.992 ± 2.731
		4th	−3.047 ± 5.706	−4.058 ± 6.609	−6.737 ± 3.793
Ankle	Right	1st	−4.972 ± 2.653	−4.921 ± 5.064	−1.508 ± 1.850
		2nd	12.062 ± 3.782	12.729 ± 5.017	15.308 ± 2.571
		3rd	−17.960 ± 4.849	−16.536 ± 4.416	−15.093 ± 5.363
		4th	6.737 ± 1.818	6.375 ± 3.692	8.952 ± 2.739
	Left	1st	−4.132 ± 4.560	−4.668 ± 3.645	−0.024 ± 3.228
		2nd	12.731 ± 3.899	12.659 ± 3.466	14.982 ± 2.575
		3rd	−16.066 ± 5.241	−15.946 ± 4.432	−14.921 ± 6.725
		4th	6.850 ± 3.128	6.568 ± 2.007	8.673 ± 2.700

Bold: significant differences with *P* < 0.05.

**Table 3:** Joint kinematic peak values (mean) of gait in the frontal plane, during the second and third trimesters of pregnancy (*N* = 22) and nulliparous controls (*N* = 12). Units (mean ± sd) are in degrees.

Joint	Side	Peak	Second trimester	Third trimester	Nonpregnant
Hip	Right	1st	**11.681 ± 3.705**	**10.282 ± 3.346**	**14.805 ± 3.808**
		2nd	−7.774 ± 4.454	−8.946 ± 3.966	−9.142 ± 2.998
	Left	1st	9.553 ± 4.031	11.086 ± 2.687	14.403 ± 3.183
		2nd	−9.821 ± 4.195	−7.920 ± 3.534	−9.768 ± 5.746
Ankle	Right	1st	−5.117 ± 3.646	−5.479 ± 6.513	−3.825 ± 2.401
		2nd	8.220 ± 5.194	8.020 ± 7.902	11.465 ± 4.102
		3rd	−0.177 ± 4.074	−0.532 ± 6.053	0.661 ± 3.896
		4th	4.455 ± 4.198	3.927 ± 4.973	5.089 ± 2.982
	Left	1st	−4.662 ± 2.317	−5.253 ± 2.202	−4.367 ± 2.040
		2nd	9.271 ± 5.613	8.808 ± 4.445	11.214 ± 3.089
		3rd	0.298 ± 3.894	0.276 ± 3.205	1.350 ± 3.711
		4th	4.594 ± 2.869	4.183 ± 3.597	6.525 ± 3.369

Bold: significant differences with *P* < 0.05.

**Table 4:** Joint kinematic peak values (mean) of gait in the transverse plane, during the second and third trimesters of pregnancy (*N* = 22) and nulliparous controls (*N* = 12). Units (mean ± sd) are in degrees.

Joint	Side	Peak	Second trimester	Third trimester	Nonpregnant
Ankle	Right	1st	−17.839 ± 8.948	−19.745 ± 7.116	−18.654 ± 5.926
		2nd	−20.365 ± 10.228	−21.871 ± 7.276	−20.277 ± 6.903
	Left	1st	−17.611 ± 6.492	−19.253 ± 9.174	−22.699 ± 5.876
		2nd	−20.951 ± 6.242	−21.670 ± 7.943	−21.050 ± 7.410
Hip	Right	1st	8.932 ± 7.699	8.083 ± 6.416	11.250 ± 5.580
		2nd	−10.377 ± 7.711	−9.104 ± 6.871	−8.269 ± 5.824
	Left	1st	7.908 ± 7.948	10.132 ± 6.534	12.355 ± 8.342
		2nd	−10.099 ± 7.887	−7.751 ± 5.256	−5.350 ± 5.965
